# Analysis of Random Lasing in Human Blood

**DOI:** 10.3390/bios14090441

**Published:** 2024-09-13

**Authors:** Sergio de Armas-Rillo, Beatriz Abdul-Jalbar, Josmar Salas-Hernández, Jose María Raya-Sánchez, Tomás González-Hernández, Fernando Lahoz

**Affiliations:** 1 Departamento de Física, Instituto Universitario de Estudios Avanzados en Física Atómica, Molecular y Fotónica (IUdEA), Universidad de La Laguna (ULL), 38206 Santa Cruz de Tenerife, Spain; 2 Departamento de Matemáticas, Estadística e Investigación Operativa, Universidad de La Laguna (ULL), 38206 Santa Cruz de Tenerife, Spain; babdul@ull.edu.es; 3 Departamento de Ciencias Médicas Básicas, Instituto de Tecnologías Biomédicas (ITB), Universidad de La Laguna (ULL), 38071 Santa Cruz de Tenerife, Spain; jsalash@ull.es (J.S.-H.); tgonhern@ull.edu.es (T.G.-H.); 4 Laboratorio de Hematología, Servicio de Hematología y Hemoterapia, Hospital Universitario de Canarias, 38320 La Laguna, Spain; jrayasan@ull.edu.es

**Keywords:** random laser, biolasers, optical sensing, whole blood, chronic lymphocytic leukemia

## Abstract

Random lasing (RL) is an optical phenomenon that arises from the combination of light amplification with optical feedback through multiple scattering events. In this paper, we present our investigations of RL generation from human blood samples. We tested mixtures of rhodamine B dye solutions with different blood components, including platelets, lymphocytes, erythrocytes, and whole blood. Intense coherent RL was obtained in all cases at relatively low pump thresholds, except for erythrocytes. We also studied the potential of RL signal analysis for biosensing applications using blood samples from healthy individuals and patients suffering from Chronic Lymphocytic Leukemia (CLL). CLL is a blood disease characterized by a high count of lymphocytes with significant morphological changes. A statistical analysis of the RL spectra based on principal component and linear discriminant analyses was conducted for classification purposes. RL-based sample discrimination was conducted for whole blood, platelet, and lymphocyte samples, being especially successful (86.7%) for the latter. Our results highlight the potential of RL analysis as a sensing tool in blood.

## 1. Introduction

Random lasing (RL), an optical phenomenon originating from the combination of light amplification with optical feedback through multiple scattering events, has undergone significant development since its proposal almost 60 years ago [1,2]. This emission diverges from conventional lasers because the resonant element is formed by scattering surfaces randomly distributed throughout the gain medium, thus establishing an open cavity system, instead of fixed built mirrors [3,4]. It typically displays weaker directionality and monochromaticity compared to conventional lasers [5,6], but is easily achievable when efficient laser dyes are embedded in media with strong scattering strength [7,8,9,10], a common characteristic of biological environments. Thus, in recent years, RL has been observed in a number of biological media: cells (in cultures [11,12] and individual cells [13]); in insect wings [14,15,16,17]; mammal bone, fat, nerve, muscle, brain, and uterine tissue [18,19,20,21]; and, more interestingly, in human tissues: breast [22], thyroid [23], and a phantom of colorectal tumor [24].

Coherent RL is characterized by narrow (<1 nm) spikes over a broad amplified spontaneous emission background [25,26]. The resulting emission spectrum is directly dependent on the scattering strength [27] and thus encodes information about the specific resonator attributes. This, coupled with the high signal-to-noise ratio of lasing emission and the lack of need for a mirror structure, makes RL-based sensors highly promising, especially for biomedical applications. Indeed, such sensors have been proposed for dopamine and immunoglobulin detection [28,29]; for stress and changes in bone structure [30,31]; as a cell apoptosis assessment [12]; or for tracking certain mutant protein expressions in cell cultures [11]. The use of RL for differentiating healthy from cancerous tissue must be highlighted. The fundamental reason for the observed RL differences between healthy and cancerous tissue is the morphological and conformational changes induced in the tissues by the disease. This modifies the light scattering inside them, which is reflected in the RL, whether in its modal structure, number of modes, or lasing threshold. Generally, tumoral tissues are more disordered and amorphous than healthy ones [22,23,32].

Blood is an accessible biological tissue extensively used in clinical practice. It presents biomarkers for a plethora of pathologies (see for example [33,34,35,36,37,38]). A random laser whose feedback source comes from scattering in whole blood (WB) could dramatically increase the potential of this technique as a biosensor. RL has been reported using red blood cells at significantly subphysiological concentrations as a gain medium [39], while the scattering mechanism needed to achieve RL was provided by mixing the diluted red blood cells with a solution of SiO_2_ nanoparticles. However, to the best of our knowledge, no RL action has been observed with WB components as the scatterers. This could be more interesting for sensing purposes, since RL characteristics are directly influenced by the scattering centres (that is, the WB components) and, therefore, changes in any of these components are expected to have an impact on the RL signal. Aside from its sensing potentialities, RL in blood would also be interesting as a mirrorless source of laser light for imaging in biological media. Due to the ease of their construction, random lasers have been proposed as a promising family of biocompatible lasers [5,26,40].

In this paper, we present our investigations of RL generation from human blood samples. Mixtures of rhodamine B (RhB) solutions with different blood components, namely platelets, lymphocytes, and erythrocytes, as well as WB, were tested. Intense coherent RL was obtained in all cases at relatively low pump thresholds, except for erythrocytes (red blood cells, RBCs), for which no RL emission was observed.

Moreover, the potential of RL signal analysis for biosensing applications has been studied, as a proof of concept, with blood samples from healthy patients and from patients suffering from Chronic Lymphocytic Leukaemia (CLL). This condition is commonly diagnosed under two criteria: first, a count above 5 × 10^9^/L B lymphocytes in blood during three consecutive months, and the determination through flux cytometry of the clonal nature of those lymphocytes [41]. This disease also induces significant morphological changes in patients’ lymphocytes (although they are not unique to CLL, thus not being enough on their own to diagnose CLL univocally). The lymphoid elements characteristic of CLL exhibit a reduced size compared with those of healthy individuals, a high ratio of nucleus to cytoplasm, weakly basophilic agranular cytoplasm, and a rounded nucleus showing the characteristic chromatin condensation known as grumelle or Grumpecht. Physiological lymphocytes, on the other hand, have a variable nucleus/cytoplasm ratio and non-condensed, homogeneous chromatin [42,43,44]. RL data from this experiment were analyzed using multivariate statistical methods that have previously been proved to be successful in biomedical classification studies [11,18,19]. Namely, statistical models based on principal component analysis (PCA) and linear discriminant analysis (LDA) were constructed and used to study the underlying differences in the RL spectra between healthy subjects and CLL patients. The results of this RL analysis are discussed in the paper.

## 2. Materials and Methods

### 2.1. Blood and Blood Component Samples

Human blood samples (11–12 mL) from 4 patients with chronic lymphocytic leukaemia and from 4 healthy control subjects (all between 42 and 65 years old) were collected by venipuncture using a 21 G needle attached to blood collection tubes with EDTA anticoagulant and stored for not more than 120 min at 4 °C until processed. One hundred µL of whole blood was separated for subsequent RL analysis. The rest of the sample was transferred to two conical centrifuge tubes (5.5–5.9 mL/each) containing 3 mL Histopaque 1077 (Sigma-Aldrich, St. Louis, MO, USA), a solution of polysucrose and sodium diatrizoate with a density of 1.077 g/mL that allows recovery of lymphocytes and other mononuclear cells from whole blood using a density gradient.

Blood was centrifuged at 535× *g* for 30 min at room temperature (RT). The supernatant was discarded. The opaque interface containing lymphocytes and platelets was diluted in 3 mL 15% citrate–dextrose in phosphate buffer solution (ACD-PBS) and centrifuged at 500× *g* for 10 min at RT. The supernatant (supernatant #1) containing platelets was transferred to a clean tube and the pellet containing lymphocytes was resuspended in 1.5 mL ACD-PBS and centrifuged at 150× *g* for 10 min. This step was repeated twice, collecting supernatants #2 and #3 in the same tube as supernantant #1. The final pellet (purified lymphocytes) was resuspended in 200 µL ACD-PBS. The pool of supernatants was also centrifuged at 535× *g* for 300 min and the pellet containing platelets was resuspended in 50 µL ACD-PBS.

### 2.2. RL Obtention and Optical Setup

For RL analysis, WB and blood component extracts were diluted (1:1) in 10 mM RhB/EtOH to produce a final dye concentration of 5 mM. In the case of blood components, mixes were prepared in 1.5 mL Eppendorf tubes, and 20 µL of the resulting mix was poured on a cavity microscope slide, then covered with a standard glass slide. In the case of WB, since it contains clotting factors (which are absent from the blood component samples due to the extraction process [45]) and EtOH promotes coagulation [46], mixing was carried out by casting 10 µL of WB directly on the cavity slide and then adding a 10 µL drop of 10 mM RhB/EtOH. Then, the sample was immediately covered before coagulation could start.

**Figure 1:**
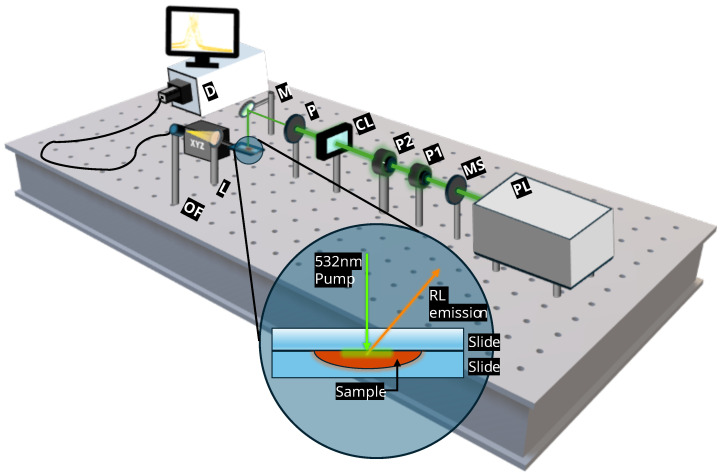
Scheme of the experimental setup used for the RL experiments. **PL**, pump laser; **MS**, mechanical shutter; **P1**, **P2**, polarizers 1 and 2; **CL**, cylindrical lens; **P**, pinhole; **M**, mirror; **L**, lens; **OF**, optical fibre, **D**, detector.

Typically, for RL to occur a high-energy pulsed laser is used as a pump source, with the excitation light well focused on the sampled area to ensure a high pump energy density and maximize gain. A frequency-doubled Nd-YAG pulsed laser (Continuum, Surelite S I-20, Milpitas, CA, USA) oscillating at 532 nm, linewidth of 0.04 nm, was the pumping source for RL, with a 20 Hz repetition rate. The duration of the excitation pulses was around 8 ns. The output beam of the laser had an energy of 2 mJ and, operating in the near field (<1 m), had a deviation from a Gaussian beam of 30%. For adequate control of pump parameters, a pinhole was placed in front of the excitation laser to obtain a homogeneous laser beam with a diameter of around 2.5 mm, while two glan-laser linear polarizers were used to control the excitation beam’s energy within a 0.5–50 µJ range. For obtaining single-shot measurements, a mechanical shutter was placed in the pump laser path. A cylindrical lens with a 100 mm focal focused the pump light in a 300 µm wide strip, while another pinhole limited the length of this strip to 4 mm. As depicted in Figure 1, the pump beam incidence was normal over the flat upper surface of the sample. RL emission was not directional, and was collected with a 60 mm lens 20 cm away from the sample at a 40° angle, which focused it into an optical fibre (Ø_Core_ 200 µm, Thorlabs M25L05, Newton, MA, USA) which brought the light to an ANDOR SR-3031-B spectrometer coupled to a Newton 970EMCCD CCD detector, resolution of 0.04 nm.

### 2.3. Statistical Analysis

The RL comparative study was performed on WB, platelet, and lymphocyte samples. For each type of sample, the RL spectra collected from all patients were subjected to a multivariate statistical analysis consisting of principal component analysis (PCA) and linear discriminant analysis (LDA). This strategy has been reported as a useful tool for classification studies in biomedical assessments [11,19,47]. Initially, PCA was performed to reduce the dimensionality of the spectral information to a smaller set of variables, retaining components with eigenvalues greater than or equal to one. Then, LDA was applied, generating a linear discriminant function based on the obtained PC scores, which was used to differentiate between healthy and CLL patients. To develop the PCA–LDA models, the datasets were randomLy divided into a training set containing 70% of the data and a validation set with the remaining 30%. The performance of the models was assessed through leave-one-out cross-validation within the training set and by classifying the samples in the validation set. All statistical analyses were performed using IBM SPSS 29 (IBM Corp., Armonk, NY, USA).

## 3. Results and Discussion

### 3.1. RL in WB and Blood Components

RL experiments were conducted in four different kinds of samples: WB, platelets, lymphocytes, and erythrocytes. All of them were mixed with a small volume of RhB/ethanol solution, as indicated in the Section 2. Emission spectra were studied as a function of increasing pump energy density (E^P^). The observed results are the following.

#### 3.1.1. Whole Blood

**Figure 2:**
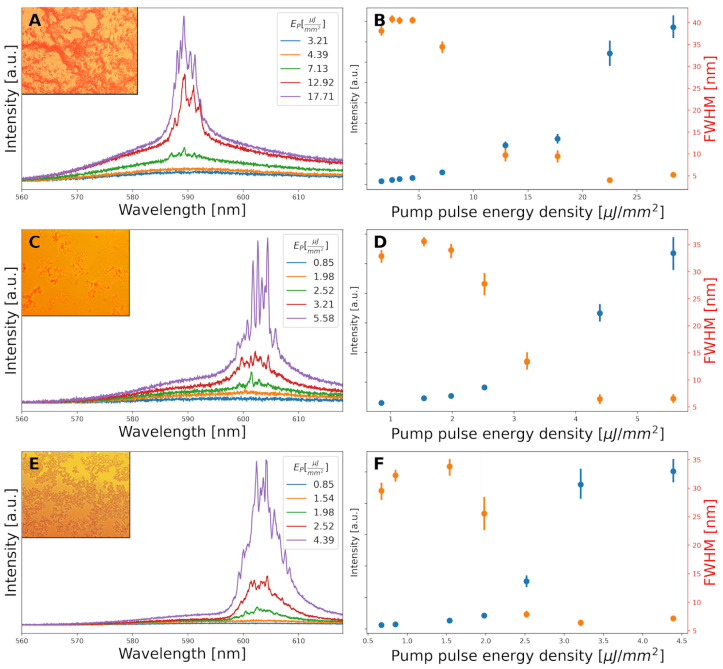
Evolution of emission spectra (left panels: **A**,**C**,**E**), and emission intensity and FWHM (right panels: **B**,**D**,**F**) as a function of increasing pump energy density for WB, platelets, and lymphocytes, respectively. Insets: optical microscopy images of the samples.

Thin samples of 5 mM RhB/EtOH+WB were interrogated with a 532 nm pulsed laser source at different pump energy densities (E_P_). At low pumping energy densities, such samples showed a broad spontaneous fluorescence emission spectrum, centered at 585 nm with an FWHM of 35–40 nm. As the pumping energy density increased the emission intensity did too, linearly, while maintaining its shape. However, when E_P_ was set above 7 µJ/mm^2^, a series of very narrow spikes with an FWHM below 0.5 nm started to appear. As the pumping energy increased further, the spectra dramatically narrowed while always maintaining the presence of narrow spikes superimposed to the luminescence. At the same time, the emission intensity started to deviate from the linear relationship with E_P_ that was following so far. Figure 2A,B illustrate the described changes in the spectra with increasing pumping energy densities. The values for panel B come from 50 different shots under the same E_P_. The spectral position and relative intensity of the spikes may change from one excitation shot to another. The plot of the FWHM of the emitting band shows that it drops to around 5 nm at a high excitation energy. This value is larger than that of the single spikes and is due to the superposition of a large set of narrow peaks. All of these, plus the spatial dependence of RL modes, are the typical characteristics of coherent RL emission, which appeared above a RL threshold of around 7 µJ/mm^2^. In order to discard some kind of self-emission from the fluorescent elements that are naturally present in blood, undyed samples were explored in the same manner, and they did not exhibit emission altogether.

As mentioned in the Section 2, WB tends to coagulate when mixed with ethanol. Our sample preparation method partially prevents that process, but cell aggregates, mainly containing RBC and platelets surrounded by plasma with sparse, free flowing cells, are still evident. This makes WB samples inhomogeneous (see inset of Figure 2A). This inhomogeneity, apparent even to the naked eye, is reflected in the RL threshold parameter, which varies noticeably when different sample regions are pumped. Indeed, we found sample regions where no RL emission was detected even under high E_P_. An RL threshold of around 7 µJ/mm^2^ is an average value of the sample regions with a bright RL emission.

#### 3.1.2. Platelets

The optical measurements were carried out as described for WB testing. A sudden increase in the emission intensity, together with the appearance of a set of narrow peaks superposed to the RhB broad photoluminescence band, was detected above a certain pumping energy density. RL in samples of isolated platelets occurred at a threshold E_P_ of around 2.3 µJ/mm^2^. This parameter was spatially homogeneous across the sample.

The observed RL modes were confined in the spectral region between 595 and 607 nm. This spectral region is redshifted by around 15 nm as compared to the RL signal of WB. The reason for this spectral difference is thought to be related to different reabsorption losses, associated with erythrocytes in WB, and also with different kinds and concentration of scatters. The causes for the relative redshift (or blueshift) of RL have been described before [26]. It is attributed mainly to changes in the media scatterers (unless the fluorescent properties of the dye are chemically or physically modified), with an increase in their concentration linked to redshift. The secondary inner filter effect gains relevance as the dwell time of the photons in the media increases due to the increased scattering [48]. WB has many more scattering structures, yet the observed effect is that of a relative blueshift of the RL from WB compared to platelets. There is not a clear reason for relative blueshift in RL systems in general [27,49,50]. However, it has been reported that an increase in non-emissive absorbers leads to the reduction in the RL wavelength, reducing the light path in the media in a reversal of the previous situation [51]. The presence of red blood cells in WB in contrast to the isolated platelets, plus the absence of saturation effects, supports this latter interpretation.

#### 3.1.3. Lymphocytes

Some laser-like spikes started appearing at E_P_ values around 2 µJ/mm^2^, a value very similar to that of platelets. These narrow peaks appeared superimposed on the typical spontaneous luminescence between 596 and 610 nm, similar to platelets, and quickly became dominant, making the fluorescence emission of the sample negligible.

There was no sign of the inhomogeneity of WB+EtOH samples. RL was present in practically the total area of the sample (avoiding the edges due to possible uncontrolled refraction and reflection effects from the cover faces), and the threshold of this RL was also constant, within the experimental accuracy.

RL showed up in a significantly different spectral position than in WB, redshifted by more than 15 nm. Also, the spectral band in which the RL modes appear in not exactly the same as the one found in platelets, although very close. While the shift from WB is expected for the same reasons as described for platelets, the spectral response of platelets and lymphocytes is supposed to be the same for visible light. This shift is also much more subtle than from WB: the latter’s is 15 nm compared to the former’s 1–2 nm. Platelets are morphologically different from lymphocytes, especially in size: 5 µm for the former and 20 µm for the latter. Also, the physiological concentration of platelets in a healthy human ranges between 150 and 450 × 10^9^/L compared to the 1–4.8 × 10^9^/L of lymphocytes. Although these are not the concentrations that our samples have, they depend on the absolute amount of each blood component in the original sample. All the other experimental parameters being the same, a higher original concentration of a component will result in a more concentrated sample. Since there are no absorbing non-fluorescent elements, an increase in the number of scatterers would result in a relative redshift of the emission [48,52]. At the same time, platelets are significantly smaller and have fewer intracellular structures than lymphocytes, which limits the effects of their higher concentration in the overall scattering strength of the sample, hence the reduced spectral shift between the two types of blood components.

#### 3.1.4. Erythrocytes

RBCs have an absorption peak at 540 nm [53], which coincides with the emission peak of RhB in EtOH, but also strong baseline absorption throughout the visible spectrum. This produces important optical losses in the spectral range of the dye emission, hindering RL action. Indeed, under the same experimental conditions as the rest of the studied components, no RL was observed in RBCs, even at very high E_P_ (>150 µJ/mm^2^). However, the fact that WB showed RL despite the important presence of erythrocytes points to there not being any physical reason that would prevent RL from scattering in red blood cells, if a different dye with bright emission beyond the absorption bands of RBD was used.

In order to rule out optical feedback coming from anything other than the cells and structures present in blood, the emission of a blank sample consisting of 5 mM RhB/EtOH was studied under increasing energies of the pumping 532 nm laser. At low energies, the samples exhibited the typical spontaneous fluorescence spectra of RhB, with a broad band centred at 595 nm with a full width at half maximum (FWHM) of around 60 nm. With growing pump powers, the emission shifted its shape, narrowing to an FWHM of 15 nm and peaking around 610 nm. Moreover, when the pump power crossed a certain value, the emission intensity started to increase dramatically. This emission has all the characteristics of amplified spontaneous emission (ASE), and none of coherent RL. This confirms that the emission behaviour and spectral structures detected in the experiments are due to the presence of the scatterers added by the different studied samples: the scattering of the light emitted by the RhB molecules is produced by the blood cells and other components present in them.

### 3.2. Observational Case Study: RL Characteristics of CLL Patients

As an example of the capabilities of RL as a sensing tool in blood, a preliminary comparative study was carried out between patients of CLL and healthy subjects. Blood samples needed to be extracted and analyzed as soon as possible to avoid cell degradation. All the RL measurements were conducted within 48 h from extraction.

**Figure 3:**
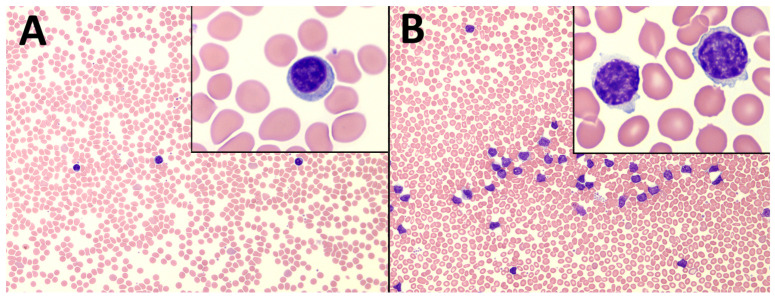
Microscopy images of lymphocytes after May–Grünwald–Giemsa dyeing from (**A**) healthy human and (**B**) CLL patient. In purple, lymphocytes; RBC in pale red. Main figures, 20× magnification, insets, 100×.

CLL was selected to test the classification power of the RL in blood because it has a profound influence on the number and morphology of lymphocytes, which, in turn, is expected to have an impact on the RL signal from blood. Indeed, as has already been mentioned in the Section 1, the most noticeable effects of the CLL are present in lymphocytes, see also Figure 3.

The RL comparison study was performed on WB, platelet, and lymphocyte samples. First, the RL thresholds were determined. The measured threshold values were within the experimental uncertainty of this parameter for each sample type. That is, there were no significant differences in the threshold for the same blood component between healthy and CLL individuals. Consequently, it could not be used as a criterion for classification.

**Figure 4:**
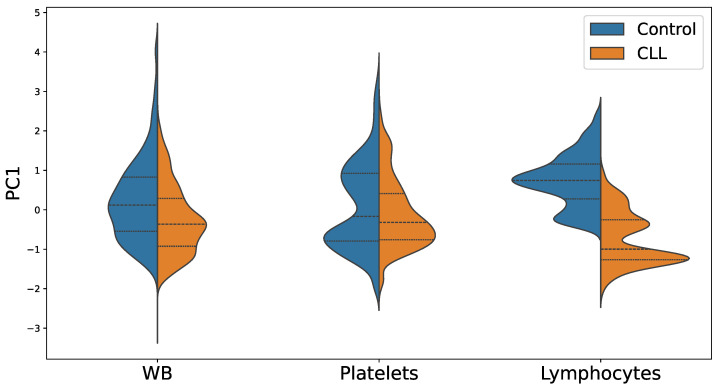
Distribution of PC1 scores for CLL and control groups for (**A**) WB, (**B**) platelet, and (**C**) lymphocyte studies.

Then, the emission of WB, platelet, and lymphocyte samples were explored by pumping different areas of them at a pump power well above the RL threshold (a 41.7 µJ/mm^2^ pump energy density was selected for WB and 13.3 µJ/mm^2^ for both platelets and lymphocytes). No photobleaching of the RL emission was observed under these excitation conditions throughout the study. For each sample, 10 different areas were interrogated, and in each area 50 pump pulses were recorded, since RL peaks at slightly different spectral positions and intensities may be detected under different excitation pump pulses. Consequently, 500 RL spectra were collected per component. As mentioned in the Section 2, the acquired spectra were subjected to a multivariate statistical analysis. First, the high dimensionality of the spectra dataset was reduced with PCA. Figure 4 shows the PC1 score distribution of CLL and control patients for WB, platelet, and lymphocyte samples. The distribution strongly overlaps for WB and platelets. However, a clear separation between CLL and control groups can be observed already from the PC1 distribution in lymphocyte samples. The fact that lymphocyte cell samples appear distinct using only PCA, while the other two types of samples do not, appears as a sound result, since CLL disease produces significant morphological changes in patients’ lymphocytes.

**Figure 5:**
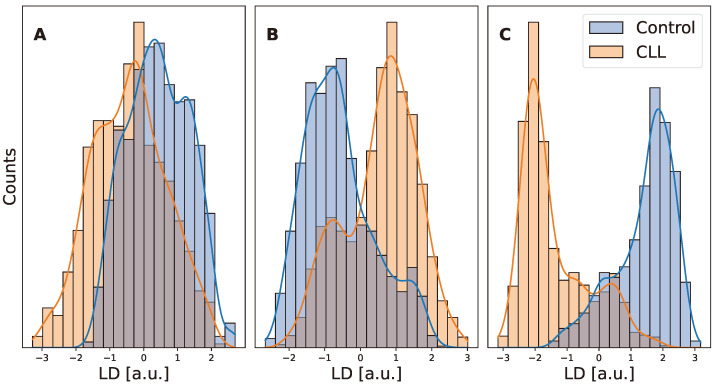
Histograms of the linear discriminant function of CLL and control groups for WB, platelet, and lymphocyte studies.

In order to improve the discrimination power of the multivariate statistical analysis, linear discriminant analysis was subsequently applied using the PCs obtained in the previous statistical step as input values. Since there are only two classification groups (control and CLL), one linear discriminant function (LD) was generated. The LD distribution of CLL differed from that of controls for the three types of samples under study, see Figure 5. Moreover, the mean values (and standard deviations) of the LD scores of CLL and control groups for the WB samples are −0.44 ± 1.09 and 0.42 ± 0.9, respectively. In platelets, they were 0.54 ± 1.03 and −0.56 ± 1.0, reducing the overlap between LD scores. As expected, the highest differentiation is observed in lymphocytes, −1.38 ± 1.07 and 1.39 ± 0.93 for CLL and controls, respectively.

As was mentioned before, the model was built using a randomLy selected training set consisting of 70% of the original dataset. A leave-one-out cross-validation technique was applied to the model. In this procedure, one spectrum is left out of the dataset and the model is rebuilt. Then, the removed spectrum is classified in the new model. The performance of the model was evaluated further by classifying the remaining 30% of the spectra in the validation set. The results are given in Table 1. The most accurate cross-validation and validation set rates are obtained for the lymphocyte samples, 87.1% and 86.7%, respectively.

This result could be expected: the clonal B lymphocytes that are the hallmark of CLL are smaller, more irregular than their counterparts in healthy humans, and present significant chromatin aggregates in their nuclei, increasing the refractive index contrasts that light finds when travelling through their samples. All these factors change the scattering characteristics of the samples and therefore affect the RL coming from them. Platelets, although they are not pathological, when far from the onset of the disease are affected by the obstruction in the bone marrow caused by the overgeneration of pathological lymphocytes. Under these conditions, platelets of CLL patients in medium–advanced stages of the disease are smaller and in lower numbers than in healthy individuals [54]. These changes are also true for WB, where we find both lymphocytes and platelets. However, classification using RL from WB proves difficult for two reasons. First, the relative weight of both platelets and lymphocytes to the scattering of WB is much lower, due to their reduced concentrations compared to the lymphocyte or platelet mixtures after extraction. Second, dyed WB samples are very inhomogeneous, which renders the analysis of the RL signal more difficult.

## 4. Conclusions

Coherent RL emission was obtained in human whole blood mixed with a solution of RhB in ethanol. Additionally, RL was also detected in blood components, namely, platelet- and lymphocyte-enriched extracts. In all the cases, the typical characteristics of coherent RL were observed. Under low pump energy density excitation, the broad emission band associated with the RhB dye molecules was recorded. However, above a certain pump energy density threshold, the emission intensity suddenly increased in a superlinear way, and a set of narrow emission lines (<1 nm) appeared superposed on the broad fluorescence band, indicating that RL emission arises from the blood samples. With RhB dye molecules providing gain, the blood cells were responsible for the light scattering events that produce the optical feedback mechanism. The measured RL threshold values were around 7 µJ/mm^2^, 2.3 µJ/mm^2^, and 2.0 µJ/mm^2^ for WB, platelets, and lymphocytes, respectively.

As a proof of concept of the capabilities of RL as a sensor in blood, a small comparative study of patients with Chronic Lymphocytic Leukaemia was carried out. WB samples were collected from CLL patients and healthy controls, and platelets and lymphocytes were also isolated from the blood extractions. All the samples were interrogated with the pump laser at energies above the RL threshold, recording five hundred RL spectra for each subject. The RL dataset was then subjected to a multivariate statistical analysis based on PCA and LDA. A classification model was built for WB and each blood component. Lymphocyte LD scores of CLL patients were clearly differentiated from those of controls (−1.38 ± 1.07 vs. 1.39 ± 0.93), and the model classified the spectra from the validation set with 86.7% accuracy. The model also was able to discriminate between groups in the platelet (71.2% success rate) and WB samples (67.2%).

A high-power pulsed laser is required as the optical pump source in order to obtain RL emission. However, nowadays there exist a large variety of commercially available compact Neodymium-doped pulsed lasers that could be implemented for this application. At the same time, the followed procedure did not require tailored materials or reactives, since the intrinsic scattering of the studied samples is enough for providing lasing feedback. Taken together, our results indicate that RL is a sensing tool in blood with potential application as a complementary diagnostic test for blood diseases. We are aware of the relatively low sample size of the study and further investigations on a larger number of patients would be desirable. However, we believe that the presented results may assist as a proof of concept of the possibilities of RL as a sensing tool in blood. We sincerely hope that this study will encourage further research in this field from the scientific community.

## Figures and Tables

**Table 1:** Cross-validation and validation set prediction scores of the LDA models for the RL of WB, platelets, and lymphocytes of CLL patients and control subjects.

			Predicted Group (%)		
			**Control**	**CLL**	**Overall Accuracy (%)**
**WB**	Training set cross-validation	Control	65.3	34.7	65.6
		CLL	34.0	66.0	
	Validation set	Control	**66.6**	33.4	**67.2**
		CLL	32.2	**67.8**	
**Platelets**	Training set cross-validation	Control	74.6	25.4	73.9
		CLL	26.8	73.2	
	Validation set	Control	**71.1**	28.9	**71.2**
		CLL	28.7	**71.3**	
**Lymphocytes**	Training set cross-validation	Control	89.7	10.3	87.1
		CLL	15.5	84.5	
	Validation set	Control	**87.8**	12.2	**86.7**
		CLL	14.3	**85.7**	

## Data Availability

The data presented in this study are openly available in Zenodo at https://zenodo.org/doi/10.5281/zenodo.13260378 (accessed on 31 August 2024).

## Abbreviations

The following abbreviations are used in this manuscript:

RL	Random lasing
WB	Whole blood
CLL	Cronic lymphocytic leukaemia
PCA	Principal component analysis
LDA	Linear discriminant analysis
RhB	Rhodamine B
EtOH	Ethanol
RBC	Red blood cells
